# Dietary Vitamin K Intake Is Associated with Cognition and Behaviour among Geriatric Patients: The CLIP Study

**DOI:** 10.3390/nu7085306

**Published:** 2015-08-12

**Authors:** Justine Chouet, Guylaine Ferland, Catherine Féart, Yves Rolland, Nancy Presse, Kariane Boucher, Pascale Barberger-Gateau, Olivier Beauchet, Cedric Annweiler

**Affiliations:** 1 Department of Neuroscience, Division of Geriatric Medicine, Angers University Hospital; Angers University Memory Clinic; UPRES EA 4638, University of Angers, UNAM, Angers F-49933, France; E-Mails: justine.chouet@gmail.com (J.C.); OlBeauchet@chu-angers.fr (O.B.); 2 Centre de recherche, Institut Universitaire de Gériatrie de Montréal, Montréal, QC H3W 1W5, Canada; E-Mails: guylaine.ferland@umontreal.ca (G.F.); nancy.presse@umontreal.ca (N.P.); kariane.boucher@gmail.com (K.B.); 3 Université Bordeaux, ISPED, Centre INSERM U897-Epidemiologie-Biostatistique, Bordeaux F-33000, France; E-Mails: catherine.feart@isped.u-bordeaux2.fr (C.F.); Pascale.Barberger-Gateau@isped.u-bordeaux2.fr (P.B.-G.); 4 INSERM, ISPED, INSERM U897-Epidemiologie-Biostatistique, Bordeaux F-33000, France; 5 Department of Geriatric Medicine, Institut du Vieillissement, University Hospital; INSERM U1027, Toulouse F-31400, France; E-Mail: rolland.y@chu-toulouse.fr; 6 Robarts Research Institute, Department of Medical Biophysics, Schulich School of Medicine and Dentistry, the University of Western Ontario, London, ON N6A 5B7, Canada

**Keywords:** cognition, behavior, diet, older adults, vitamin K

## Abstract

Our objective was to determine whether dietary vitamin K intake was associated with cognition and behavior among older adults. 192 consecutive participants ≥65 years, recruited in the cross-sectional CLIP (Cognition and LIPophilic vitamins) study, were separated into two groups according to the tertiles of dietary phylloquinone intake (*i.e.*, lowest third below 207 µg/day *versus* the other two thirds combined). Daily dietary phylloquinone intake was estimated from 50-item interviewer-administered food frequency questionnaire. Cognition was assessed with Mini-Mental State Examination (MMSE); behaviour with Frontotemporal Behavioral Rating Scale (FBRS). Age, gender, social problems, education, body mass index (BMI), comorbidities, history of stroke, use vitamin K antagonists, inadequate fatty fish intake, serum thyroid-stimulating hormone (TSH), vitamin B12, albumin, and estimated glomerular filtration rate were used as confounders. Compared to participants in the lowest third of dietary phylloquinone intake (*n* = 64), those with higher intake had higher (*i.e.*, better) mean MMSE score (22.0 ± 5.7 *versus* 19.9 ± 6.2, *p* = 0.024) and lower (*i.e.*, better) FBRS score (1.5 ± 1.2 *versus* 1.9 ± 1.3, *p* = 0.042). In multivariate linear regressions, log dietary phylloquinone intake was positively associated with MMSE score (adjusted β = 1.66, *p* = 0.013) and inversely associated with FBRS score (adjusted β = −0.33, *p* = 0.037). Specifically, log dietary phylloquinone intake correlated negatively with FBRS subscore of physical neglect (*r* = −0.24, *p* = 0.001). Higher dietary phylloquinone intake was associated with better cognition and behavior among older adults.

## 1. Introduction

Vitamin K is a fat-soluble substance found mainly in green vegetables and some vegetable oils. Vitamin K is classically known for its role as a coenzyme in the biological activation of seven proteins involved in blood coagulation [1]. Recently, a role for vitamin K has been demonstrated in target organs, such as the central nervous system (CNS) [2,3,4,5,6,7,8,9,10]. At the neuronal level, vitamin K is involved in the synthesis of sphingolipids—a major constituent of the myelin sheath and neuronal membranes—and the biological activation of vitamin K-dependent proteins (VKDPs) involved in neuronal physiology and survival [2,3]. Insufficient levels of vitamin K may, instead, cause neuropathological dysfunction [4]. Accordingly, an epidemiological study has reported a significant association between higher serum phylloquinone concentration (*i.e.*, vitamin K_1_) and better verbal episodic memory performance in older adults [5]. Similarly, we found that the use of vitamin K antagonists (VKAs), which deplete the active form of vitamin K, was associated with cognitive impairment [6] and a lower volume of gray matter in the hippocampus [7] among geriatric patients. Taken together, these results suggest the importance of adequate (*i.e.*, high enough) vitamin K levels for optimal cognition in older adults [11]. To date, no randomized controlled trial has explored the benefits of vitamin K supplementation to maintain or improve cognition and related behavioral disorders in older adults. Before conducting such an expensive and time-consuming trial, it seems important and contributory to determine whether the intake of vitamin K relates to cognitive and behavioral performance in older adults.

We had the opportunity to examine the association between dietary vitamin K intake and cognitive behavioral performance in a sample of geriatric patients: the CLIP (Cognition and LIPophilic vitamins) cohort. Our objective was to determine whether dietary vitamin K intake was associated with cognitive and behavioral performance among geriatric patients.

## 2. Materials and Methods

### 2.1. Participants

We studied in- and outpatients aged 65 and over, consecutively recruited in the CLIP study. The CLIP study is an observational cross-sectional study designed to examine the relationships between neurocognition and lipophilic vitamins among all patients consecutively hospitalized or seen in consultation in the geriatric acute care unit of the University Hospital of Angers, France, from February to April 2014. After giving their informed consent for research, included participants received a full medical examination consisting of structured questionnaires, a standardized clinical examination, and a blood test. The study was conducted in accordance with the ethical standards set forth in the Helsinki Declaration (1983). The entire study protocol was approved by the local ethical committee (No. 2014-33).

### 2.2. Explanatory Variable: Dietary Intake of Vitamin K

Dietary vitamin K intake was estimated from a semi-quantitative food frequency questionnaire (FFQ) [12]. The 50-item FFQ used here was specifically designed to determine the daily dietary phylloquinone intake during the previous 12 months. It comprises 50 food items identified as important contributors to phylloquinone intake (e.g., spinach, iceberg lettuce, collards, and broccoli) and items with very high phylloquinone content (≥500 µg/usual portion). The FFQ was interviewer-administered in 30 min by questioning the patients and/or their relatives, when applicable. Estimated phylloquinone intake was calculated for each food item by multiplying the amount of phylloquinone for that food item by the selected frequency and serving size (calculated as 0.5 for smaller than, and 1.5 for larger than the suggested portion), and all values were added to provide an estimate in µg/day of each participant’s daily phylloquinone intake. The vitamin K FFQ shows good relative agreement with five-day food records (κ = 0.60, *p* < 0.001) [12]. In the present analysis, the participants were categorized into two groups based on the first tertile of the dietary phylloquinone intake: those in the lowest third of dietary phylloquinone intake below 207 µg/day, and those in the other two thirds combined of dietary phylloquinone intake above 207 µg/day.

### 2.3. Dependent Variables: Neuropsychiatric Measures

Cognition was assessed by one neuropsychologist blinded from participants’ vitamin K intake using the Mini-Mental State Examination (MMSE) [13], in the absence of delirium identified with the Confusion Assessment Method [14]. The MMSE is a well-established measure of global cognitive performance in older adults composed of five sections (orientation, registration, attention-calculation, recall, and language). Scores range between 30 and 0 (worst). It shows good test-retest and inter-rater reliability and performs satisfactorily against more detailed measures of cognitive function [13].

Behavior was assessed at the same time as the MMSE using the Frontotemporal Behavioral Rating Scale (FBRS) [15]. The FBRS indicates the presence of symptoms of four domains of behavioral disturbances (*i.e.*, self-control disorder, physical neglect, mood disorders and loss of general interest). Each domain is scored 1 if at least 1 symptom is present, and 0 if no symptoms are present. The total FBRS score ranges from 0 (normal) to 4 (worst). The FBRS is easy to perform and has demonstrated good test-retest reliability [15].

### 2.4. Covariables

Age, gender, social problems, education level, body mass index, comorbidity burden, history of stroke, use of VKAs, regular fatty fish and eggs intakes, serum concentrations of thyroid-stimulating hormone (TSH), vitamin B12, albumin, and estimated glomerular filtration rate (*i.e.*, creatinine clearance, eGFR) are covariates related to diet and cognitive behavioral performance and were used as potential confounders. Evaluation of education level was based on self-report. Participants who passed at least the Elementary School Recognition Certificate were considered to have high education level. Social problems were defined by a geriatrician (yes/no) as the existence of social and/or familial isolation with consequent difficulties to stay in the usual place of life. Comorbidity burden was estimated with the Cumulative Illness Rating Scale-Geriatrics score (CIRS-G) (range 0–60, worst) [16]. History of stroke was sought by questioning the patients, the family physicians, and the patients’ files. Stroke was defined according to the World Health Organization criteria as rapidly developed signs of focal or global disturbance of cerebral function lasting longer than 24 h, with no apparent nonvascular cause [17]. In case of clinical suspicion, computed tomography or magnetic resonance imaging scan was necessary to confirm the diagnosis and to distinguish among ischemic stroke and intracranial hemorrhage. The regular use of VKAs was systematically noted from the primary care physicians’ prescriptions and sought by questioning the patients and their relatives, whatever the type of VKA used (*i.e.*, warfarin, acenocoumarol, or fluindione), the indication, length of treatment, and history of international normalized ratio (INR). The regular intake of fatty fish and eggs (*i.e.*, dietary sources of vitamin D and omega-3 polyunsaturated fatty acids (*n*-3 PUFAs), both linked to cognition) was sought using a standardized question: “Do you eat fatty fish at least once a week and/or eggs several times per week?” and was coded as low (*i.e.*, answer “No”) or adequate (*i.e.*, answer “Yes”), as previously published [18]. Finally, the serum concentrations of TSH, vitamin B12, albumin and creatinine were measured using automated standard laboratory methods at the University Hospital of Angers, France, and eGFR was calculated using the Cockcroft-Gault formula (((140 – age _years_) × weight _kg_/creatinine _µmol/L_) × 1.04 for females, and ×1.25 for males).

### 2.5. Statistical Analysis

The participants’ characteristics were summarized using means and standard deviations or frequencies and percentages, as appropriate. Statistics were performed on logarithmically-transformed values for the dietary phylloquinone intake to improve the symmetry of the non-Gaussian distribution. Firstly, comparisons of participants’ characteristics according to the dietary phylloquinone intake (*i.e.*, the lowest third *versus* the other two thirds combined) were performed using Student’s *t*-test or the Chi-square test, as appropriate. Secondly, the mean difference of neuropsychiatric scores was calculated between participants in the lowest third *versus* the other two thirds combined of dietary phylloquinone intake. Thirdly, multiple linear regressions were used to examine the associations of log dietary phylloquinone intake (independent variable) with the MMSE score and the FBRS score (dependent variables), while adjusting for potential confounders. Separate analyses were conducted for each dependent variable. Finally, we examined the correlation between log dietary phylloquinone intake and each subscore of the FBRS. Lastly, we performed a sensitivity analysis after removing the participants who used VKA (*n* = 31). *p-*Values < 0.05 were considered significant. All statistics were performed using SPSS (v19.0, IBM corporation, Chicago, IL, USA) and RevMan (v5.1, Nordic Cochrane Centre, Copenhagen, Denmark).

## 3. Results

**Figure 1:**
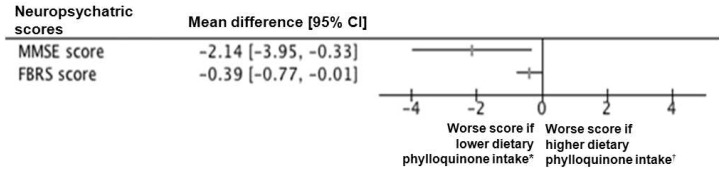
Forest plot for the mean difference of neuropsychiatric scores according to the dietary phylloquinone intake. Horizontal lines correspond to the 95% confidence interval (CI). The vertical line corresponds to a mean difference of 0.00, equivalent to no between-group difference. FBRS: Frontotemporal Behavioral Rating Scale; MMSE: Mini-Mental State Examination; *: lowest third of dietary phylloquinone intake below 206.97 µg/day; ^†^: two other thirds combined of dietary phylloquinone intake.

Among 192 included participants (mean ± standard deviation, 82.8 ± 7.1years; 62.5% female; 63.4% inpatient), the mean dietary phylloquinone intake estimated using a validated food frequency questionnaire (FFQ) [12] was 319.9 ± 196.3 µg/day. Sixty-four participants were in the lowest third of dietary phylloquinone intake, *i.e.*, below 207 µg/day (Table 1). The mean Mini-Mental State Examination score (MMSE) score was 21.4 ± 5.9 [13], and the mean Frontotemporal Behavioral Rating Scale (FBRS) score was 1.6 ± 1.2 [15]. Participants in the other two thirds combined of dietary phylloquinone intake had better MMSE and FBRS scores than those in the lowest third (respectively, 22.0 ± 5.7 *versus* 19.9 ± 6.2 with *p* = 0.024, and 1.5 ± 1.2 *versus* 1.9 ± 1.3 with *p* = 0.042) (Table 1). The between-group mean difference of MMSE score was −2.14 (95% confidence interval (CI): −3.95; −0.33), and that of FBRS score was 0.39 (95% CI: 0.01; 0.77) (Figure 1).

**Table 1:** Baseline characteristics of 192 participants by dietary phylloquinone intake.

	Total Cohort (*n* = 192)	Dietary Phylloquinone Intake *		*p*-Value
		<207 µg/day (*n* = 64)	≥207 µg/day (*n* = 128)	
**Demographical measures**				
Age, years	82.8 ± 7.1	83.7 ± 5.9	82.4 ± 7.7	0.227
Female gender, *n* (%)	120 (62.5)	43 (67.2)	77 (60.2)	0.343
Social problems, *n* (%)	21 (10.9)	10 (15.6)	11 (8.6)	0.141
High education level ^†^, *n* (%)	152 (79.2)	51 (79.7)	101 (78.9)	0.900
**Clinical measures**				
Body mass index, kg/m^2^	26.2 ± 5.4	25.8 ± 4.9	26.4 ± 5.6	0.492
CIRS-G score, /60	8.3 ± 4.0	9.0 ± 4.4	8.0 ± 3.8	0.118
History of stroke, *n* (%)	29 (15.2)	10 (15.6)	19 (15.0)	0.904
Use of vitamin K antagonists, *n* (%)	31 (16.1)	11 (17.2)	20 (15.6)	0.781
Dietary intake of phylloquinone, µg/day	319.9 ± 196.3	125.3 ± 52.1	417.1 ± 167.4	**<0.001**
Low dietary intake of fatty fish and eggs ^‡^, *n* (%)	23 (12.2)	9 (14.8)	14 (10.9)	0.453
**Neuropsychiatric measures**				
MMSE score, /30	21.4 ± 5.9	19.9 ± 6.2	22.0 ± 5.7	**0.024**
FBRS score, /4	1.6 ± 1.2	1.9 ± 1.3	1.5 ± 1.2	**0.042**
**Serum measures**				
TSH concentration, mIU/L	1.6 ± 1.8	1.5 ± 1.2	1.7 ± 2.0	0.440
Vitamin B12 concentration, ng/L	444.3 ± 266.2	478.5 ± 375.8	427.4 ± 190.6	0.228
Albumin concentration, g/L	34.9 ± 5.5	33.0 ± 5.7	35.9 ± 5.2	**0.001**
Estimated glomerular filtration rate, mL/min	53.7 ± 21.9	50.8 ± 18.2	55.1 ± 23.5	0.217

Data presented as mean ± standard deviation when applicable. CIRS-G: Cumulative Illness Rating Scale for Geriatrics; FBRS: Frontotemporal Behavioral Rating Scale; MMSE: Mini-Mental State Examination; TSH: thyroid stimulating hormone; *: lower dietary phylloquinone intake defined as the lowest third (*i.e.*, below 206.97 µg/day); higher dietary phylloquinone intake defined as the other two thirds combined (*i.e.*, above 206.97 µg/day); ^†^: Elementary School Recognition Certificate passed; ^‡^: Answer “No” to the question “Do you eat fatty fish at least once a week and/or eggs several times per week?”; *p*-values < 0.05 indicated in bold.

Results of multiple linear regression models were reported in Table 2. We observed a positive association between log dietary phylloquinone intake and the MMSE score (β = 1.66, *p* = 0.013), and an inverse association with the FBRS score (β = −0.33, *p* = 0.037). Higher education level and higher serum concentrations of thyroid-stimulating hormone (TSH) and albumin were also associated with higher (*i.e.*, better) MMSE score, but none was associated with the FBRS score (Table 2).

Finally, Table 3 reports the correlations between the log dietary phylloquinone intake and the subscores of the FBRS. Log dietary intake of phylloquinone correlated negatively with the subscore of physical neglect (*r* = −0.24, *p* = 0.001), but not with the subscores of self-control disorders (*p* = 0.060), mood disorders (*p* = 0.196) and loss of general interest (*p* = 0.699).

**Table 2:** Fully adjusted linear regressions examining the association between the dietary phylloquinone intake (independent variable) and the MMSE and FBRS scores * (dependent variables), adjusted for potential confounders (*n* = 192).

	Neuropsychiatric Measures					
	MMSE Score			FBRS Score		
	β	(95% CI)	*p*-value	β	(95% CI)	*p*-value
Log dietary phylloquinone intake	1.66	(0.36; 2.95)	0.013	−0.33	(−0.63; −0.02)	0.037
Age	−0.22	(−0.39; −0.06)	0.009	0.02	(−0.03; 0.06)	0.450
Female gender	−0.63	(−2.46; 1.19)	0.492	−0.01	(−0.47; 0.44)	0.953
Social problems	2.75	(−0.31; 5.82)	0.078	0.10	(−0.63; 0.83)	0.792
High education level ^†^	2.52	(0.27; 4.77)	**0.029**	−0.11	(−0.67; 0.45)	0.691
Body mass index	0.02	(−0.18; 0.23)	0.842	0.02	(−0.04; 0.07)	0.561
CIRS-G score	−0.14	(−0.41; 0.13)	0.312	0.03	(−0.04; 0.09)	0.441
History of stroke	−1.56	(−4.26; 1.13)	0.253	0.38	(−0.27; 1.02)	0.248
Use of vitamin K antagonists	−0.07	(−2.69; 2.56)	0.628	−0.10	(−0.75; 0.56)	0.773
Low dietary intake of fatty fish and eggs ^‡^	−0.09	(−2.83; 2.65)	0.948	0.63	(−0.06; 1.31)	0.072
TSH concentration	0.76	(0.02; 1.49)	**0.044**	0.06	(−0.13; 0.24)	0.556
Vitamin B12 concentration	−0.00	(−0.01; 0.00)	0.513	0.00	(0.00; 0.00)	0.281
Albumin concentration	0.24	(0.04; 0.43)	**0.018**	0.01	(−0.04; 0.06)	0.698
Estimated glomerular filtration rate	−0.02	(−0.07; 0.04)	0.567	−0.01	(−0.02; 0.01)	0.242

β: coefficient of regression corresponding to a change of cognitive score; CI: confidence interval; CIRS-G: Cumulative Illness Rating Scale for Geriatrics; FBRS: Frontotemporal Behavioral Rating Scale; MMSE: Mini-Mental State Examination; TSH: Thyroid Stimulating Hormone; *: Separate models were used for each dependent variable; ^†^: Elementary School Recognition Certificate passed; ^‡^: Answer “No” to the question “Do you eat fatty fish at least once a week and/or eggs several times per week?”; β significant (*i.e.*, *p* < 0.05) indicated in bold.

**Table 3:** Correlation matrix of the dietary phylloquinone intake with the subscores of the Frontotemporal Behavioural Rating Scale.

Characteristics	1.	2.	3.	4.	5.
**1. Log dietary phylloquinone intake**	-	−0.14	−0.24 **	−0.09	−0.03
**2. Self-control disorders**		-	0.16 *	0.29 ***	0.32 ***
**3. Physical neglect**			-	0.14	0.25 ***
**4. Mood disorders**				-	0.35 ***
**5. Lower general interest**					-

*: *p* < 0.05 (2-tailed); **: *p* < 0.01 (2-tailed); ***: *p* < 0.001 (2-tailed).

Lastly, the sensitivity analysis found unaltered results after removing all participants who used VKA (*n* = 31). Of the 161 remaining participants, those in the lowest third of dietary phylloquinone intake had worse MMSE score (19.9 ± 6.0 *versus* 22.4 ± 5.8, *p* = 0.016) and FBRS score (1.9 ± 1.3 *versus* 1.5 ± 1.2, *p* = 0.035) than those in the other two thirds combined. Log dietary phylloquinone intake was linearly associated with the MMSE score (β = 1.83 (95% CI: 0.46; 3.20), *p* = 0.009) and with the FBRS score (β = −0.34 (95% CI: −0.66; −0.01), *p* = 0.041). Log dietary phylloquinone intake correlated negatively with the subscores of physical neglect (*r* = −0.24, *p* = 0.003) and self-control disorders (*r* = −0.17, *p* = 0.037), but not with mood disorders (*p* = 0.127) and loss of general interest (*p* = 0.978).

## 4. Discussion

The main finding of this cross-sectional study is that, irrespective of all measured potential confounders, increased dietary phylloquinone intake was associated with better cognition and behavior among geriatric patients. Specifically, compared to those in the lowest third of dietary phylloquinone intake, participants with higher dietary phylloquinone intake had 2.1 points more on the MMSE (*i.e.*, better) and 0.4 points less on the FBRS (*i.e.*, better).

These findings are consistent with the emerging epidemiological literature on vitamin K and cognition. Specifically, previous studies have reported that the serum concentration of phylloquinone was lower among patients with Alzheimer’s disease (AD) compared to cognitively healthy controls [8], and that higher serum phylloquinone concentration was associated with better verbal episodic memory performance [5]. In line with this, we recently found that the use of VKAs, a drug class that generates a relative state of vitamin K deficiency, was associated among geriatric patients with greater prevalence of cognitive disorders [6], and with lower volume of gray matter in the brain, including in the hippocampus [7]. Here, we found no association between the use of VKAs and the global cognitive performance used as a quantitative variable. Several explanations can be proposed to account for this divergence: first, the potential lack of power of the present study that was not originally designed to assess the covariate VKA; second, the use of the cognitive performance as a continuous variable, unlike the previous study [6], which was focused on the cognitive impairment as a categorical variable; third, the lack of specification on the length of treatment and stability of the INR, which could influence the potential cognitive effect of VKAs. Of note, removing from the analysis the participants who were taking VKAs regularly did not alter our results, which strengthened the possibility of a specific link between dietary vitamin K intake and cognition. To the best of our knowledge, only two case-control studies have specifically examined the intake of phylloquinone in relation to cognition [9,10]. Shatenstein *et* *al.* found that that the mean dietary phylloquinone intake was persistently two-fold less in 36 community-dwelling patients with early-stage AD followed during 18 months in the Nutrition-Memory Study compared to 58 cognitively healthy control subjects (55.5 ± 54.3 *versus* 104.5 ± 83.7 with *p* < 0.001 at baseline) [9]. Consistently, Presse *et* *al.* found, in an additional analysis of the Nutrition-Memory Study cohort, that 31 cases with mild-to-moderate AD had lower dietary phylloquinone intake compared to 31 age- and gender-matched cognitively-healthy control subjects (37 µg/day *versus* 70 µg/day, *p* < 0.0001), even after adjusting for energy intakes (*p* = 0.0003) [10]. Additional indirect evidence stems from two cohort studies in which the high consumption of green leafy and cruciferous vegetables, rich in vitamin K, was associated with slower rates of age-related cognitive decline [19,20]. Compared to these studies, we did not use diet records to estimate the dietary phylloquinone intake, but a FFQ. Nevertheless, despite this methodological divergence, we observed that the higher the dietary phylloquinone intake, the better the cognitive performance and the lower the behavioural disorders. Specifically, we found a correlation of dietary phylloquinone intake with self-control disorders and physical neglect, but not with mood disorders and loss of interest (Table 3). In other words, the intake of phylloquinone does not appear to be related to emotional and affect regulation, but rather to behavioral disorders in relation with the cognitive sphere, which was confirmed by the finding of a direct association between dietary phylloquinone intake and MMSE score.

The mechanism linking dietary phylloquinone intake with cognition and behavior is not fully elucidated. Causality cannot be inferred from our cross-sectional study. It is possible that worse cognitive performance and greater behavioral disorders precipitate loss of functional autonomy with consequent poorer access to vitamin K-rich food. For instance, lower consumption of green vegetables, a major source of phylloquinone, has been reported in participants with AD compared to cognitively healthy participants [10]. In addition, the association of dietary phylloquinone intake with neuropsychiatric performance could be explained in a more general way by an overall healthy diet, which could also reflect generally healthy lifestyles. However, the latter assumptions should be mitigated here by the fact that the mean BMI of 26.2 kg/m^2^ in our study indicated overweight according to the National Heart, Lung, and Blood Institute [21], and because there was no BMI difference between participants in the lowest third of phylloquinone intake and those in the highest thirds ( *p* = 0.492, Table 1). Moreover the associations of dietary phylloquinone intake with the MMSE score and the FBRS score were significant even after adjustment for BMI and albumin concentration (Table 2). A scenario of reverse causation is plausible and should be considered. Evidence precisely supports a role for vitamin K in the CNS. Vitamin K modulates the synthesis and metabolism of sphingolipids, which are key players in neuronal proliferation, differentiation, senescence, cell-cell interaction, and transformation [2,3]. Recent research has linked alterations in sphingolipid metabolism to the aging process and neurodegenerative disorders such as AD [3]. In parallel, two VKDPs, Gas6 (growth arrest-specific gene 6) and protein S, are also closely associated with the CNS functioning [2,3]. Gas6 is involved in chemotaxis, mitogenesis, cell growth, and myelination, and has further been shown to rescue cortical neurons from amyloid β-induced apoptosis, a hallmark of AD [22]. Protein S offers neuronal protection during ischemic/hypoxic injury, both *in* *vivo* and *in* *vitro* [23]. Vitamin K may also protect neurons from *N*-methyl-d-aspartate-induced toxicity and apoptosis [24]. Finally, other authors have proposed that insufficient vitamin K could contribute to the pathogenesis of AD through a link to the apolipoprotein Eε4 allele, an established risk factor for AD that is also associated with lower vitamin K levels [11]. ApoE genotype was not determined in our cohort, which precludes testing this assumption. All these data suggest that the less available the vitamin K is, the less protected and effective the CNS may be, with subsequently greater risks of brain changes, cognitive decline, and behavioral disorders.

The strengths of our study include the originality of the research question on a highly common condition in older adults, the standardized collection of data from a single research center, the assessment of the dietary phylloquinone intake over preceding 12 months based on a validated tool, the assessment of both cognitive and behavioral outcomes, and the detailed description of the participants’ characteristics allowing the use of regression models to measure adjusted associations. Regardless, a number of limitations should be acknowledged. First, due to the limited number of 192 in- and outpatients, our study may lack power and the participants may be not representative of the population of all seniors. Second, our study is cross-sectional at present, which precludes inferring causality. Third, the FFQ was validated on a Canadian population [12], and it is likely that the eating habits of our elderly patients were not strictly the same as those of North Americans. Moreover, it is noticeable that FFQs tend to overestimate intakes [25,26]. Nonetheless, the FFQ used in the present study presents a very good relative agreement with results from 24 h dietary recalls in healthy older adults [12]. It is also possible that the dietary phylloquinone intake may have been poorly estimated by interviewing patients with cognitive disorders. However, to avoid this bias, we double-checked information whenever possible from formal or informal carers. Fourth, although we were able to control for important characteristics that could modify the associations, residual potential confounders such as the determination of energy and homocysteine intakes or the determination of ApoE genotype, might still be present. Fifth, limitations include the use of the MMSE and FBRS tools, which may exhibit ceiling effects and limited sensitivity to subtle abnormalities [27]. In future studies, the screening for cognitive disorders should use other outcomes, e.g., comparing episodic memory or executive functions according to dietary phylloquinone intakes.

## 5. Conclusions

In conclusion, we report a clinically and statistically significant association between increased dietary phylloquinone intake and better cognition and behavior among geriatric patients. There is a strong need for novel effective preventive and therapeutic strategies for cognitive decline and dementia. The potential role of vitamin K in the development of dementia is therefore of substantial interest. Further prospective nutrition studies and clinical trials are needed to clarify whether older adults with higher dietary vitamin K intake are less likely to experience cognitive and behavioral declines than those with lower intake, and whether enhancing vitamin K intake could improve, or prevent, this process.
